# Enhancing Tuberculosis Case-Finding: A Case of Quality Improvement Initiative in Tanzania

**DOI:** 10.3390/tropicalmed7060097

**Published:** 2022-06-09

**Authors:** Eliud Wandwalo, Deus V. Kamara, Mohammed A. Yassin, Linden Morrison, Nnamdi B. Nwaneri, Sarah Asiimwe, Sode Matiku, Riziki Kisonga, Allan Tarimo

**Affiliations:** 1 The Global Fund to Fight AIDS, Tuberculosis and Malaria, 1218 Geneva, Switzerland; eliud.wandwalo@theglobalfund.org (E.W.); mohammed.yassin@theglobalfund.org (M.A.Y.); linden.morrison@theglobalfund.org (L.M.); nnamdi.nwaneri@theglobalfund.org (N.B.N.); sarah.asiimwe@theglobalfund.org (S.A.); 2 The National TB and Leprosy Programme, Dodoma 40478, Tanzania; kisongariziki@gmail.com (R.K.); tdrallan@gmail.com (A.T.); 3 New Dimension Consulting, Dar es Salaam 14121, Tanzania; sodematiku@gmail.com

**Keywords:** TB, Tanzania, contact tracing, missing cases, QI-TB model initiative

## Abstract

Background: Tanzania is 1 of the 30 high TB burden countries and 1 of the 13 countries in which 75% of people with TB are unaccounted for and that is prioritized for the Global Fund Catalytic investment and Strategic Initiative support. Tanzania decided to strengthen its National TB Programme to find these people with TB who are unaccounted for by identifying evidence-driven innovations to deliver high-quality services and to improve the efficiency of TB case-finding. A quality improvement (QI) initiative was implemented by the National Tuberculosis and Leprosy Programme to enhance TB case-finding. The initiative involved identifying gaps in the quality of services, introducing new tools, improving the work capacity of health care workers through training and mentorship sessions, strengthening laboratory and referral services, and implementing mandatory TB screening of all patients attending health facilities. We aimed to assess the effectiveness of QI initiative to enhance TB case-findings at the health facility level. Method: A cross-sectional design, and intervention and control facilities randomly selected for an evaluation of the QI initiative were used. Twenty facilities from the Dodoma region across all health care system levels (dispensaries, health centres, and hospitals) were involved in this evaluation. The facilities were randomly divided into either the intervention or control groups at a 1:1 ratio (10 intervention and 10 control facilities). Data routinely collected from program registers from January 2016 to June 2017 were used. Result: The evaluation registered a 52% increase in TB case notification in Q1 of 2017 compared with in Q1 of 2016 and, similarly, a 52% increase in Q2 of 2017 compared with in Q2 of 2016, with 9 out of 10 intervention sites reporting increases in their quarterly TB case notifications. There were no positive changes in the ‘control facilities’ where routine services were provided, with half of the facilities showing a decrease in TB case notification from baseline. Conclusion: This QI initiative has the potential to support a long-term comprehensive approach to ending TB and to improve the quality of the foundations of the health care system. This initiative sets a reliable pace for health facilities to efficiently respond to and manage TB case-finding interventions put into action. Tanzania’s experience with implementing QI interventions could serve as a model for improving TB case notifications in other settings.

## 1. Introduction

TB is the ninth leading cause of death worldwide and the leading cause from a single infectious agent, ranking above HIV/AIDS. Over 25% of TB deaths occur in the African Region [1]. The WHO reported 5.8 million new cases in 2020 and estimated 1.32 million TB deaths globally in 2020 [2]. The 30 high TB burden countries collectively contributed to 87% of the global TB burden, where 24% of the cases were from African countries [2]. To reduce the high TB burden, countries have been implementing the WHO-recommended TB-control strategies at various time points, including the directly observed treatment short-course (DOTS), the Stop TB strategy, and the End TB strategy in 1993/1994, 2006, and 2015, respectively. However, although there has been a general trend of a decline in global TB incidence, this reduction has been very slow, particularly in the African region, because of various factors including poverty [2,3,4,5], high TB/HIV/DM comorbidities, drug-resistant TB (MDR-TB) [2,6], poor access to quality health care services [4,6,7], and low level of community awareness about TB [4,8,9,10,11]. Of the estimated 10 million people with active tuberculosis (TB) globally, three million were ‘missed’ by health systems [2]. Finding these people unaccounted for is a global health priority, and initiatives that have combined traditional TB case-finding approaches with quality improvement (QI) approaches for detecting patients with TB who are unaccounted for in health facilities and at the community level have shown success in a number of countries in Africa [12,13,14,15,16]. Tanzania is 1 of the 30 high TB burden countries [17] and 1 of the 13 countries in which 75% of people with TB are unaccounted for and that is prioritized for the Global Fund Catalytic investment [18,19]. Tanzania decided to strengthen its National TB Programme to find these people with TB who are unaccounted for by identifying evidence-driven innovations to deliver high-quality services and to improve the efficiency of TB case-finding. An assessment was undertaken in April 2016 to understand the challenges better, to identify best practices and opportunities for improving TB case-finding at the health facility level. A total of 30 health facilities in six regions were included. The assessment revealed a low TB suspicion index among health care workers, inadequate usage of diagnostic algorithms, and poor coordination of case-finding activities. Based on the assessment, four evidence-based approaches, a QI model toolkit guide, a training package, a national QI model team, data collection tools, and job aids have been developed. A national training course on QI in TB case-finding was rolled out for orientations to health facility units by a national QI model team and training of champion district mentors and facility-level TB QI focal persons. We assessed the effectiveness of QI initiatives in enhancing TB case-findings at the health facility level. In the context of health care, QI is a framework used to systematically improve the way in which care is delivered to patients, whereby the processes have characteristics that can be measured, analysed, improved, and controlled.

## 2. Methodology

### 2.1. Study Design and Setting

A cross-sectional design, and intervention and control facilities randomly selected for an evaluation of the QI initiative were used. This design was used only to gather and analyse the relevant data but did not influence actual clinical implementation in health facilities. Twenty facilities from the Dodoma region across all health care system levels (dispensaries, health centres, and hospitals) were involved in this evaluation. The facilities were randomly divided into either the intervention or control groups at a 1:1 ratio (10 intervention and 10 control facilities). The intervention facilities were from the Dodoma municipal council, and Chamwino and Mpwapwa districts, and these facilities included Dodoma Regional Hospital, Hombolo Health Centre, Makole Health Centre, Mkonze Health Centre, Mvumi Mission Hospital, Chamwino Health Centre, Mpwapwa District Hospital, Kibakwe Health Centre, Chipogoro, and Itiso Dispensaries. The control facilities were from the Bahi, Kondoa, and Kongwa districts, and these facilities included Bahi Health Centre, Kigwe Health Centre, Mundemu Health Centre, Kondoa District Hospital, Busi Health Centre, Mnenia Health Centre, Kongwa District Hospital, Kibaigwa Health Centre, Mlali Health Centre, and Hamai Health Centre. Data routinely collected from programme registers from January 2016 to June 2017 were used.

### 2.2. Intervention

The intervention facilities received close follow-up through the use of presumptive registers at all entry points, mentorship, and supervision.

### 2.3. Implementation

A QI initiative was implemented by the National Tuberculosis and Leprosy Programme to enhance TB case-finding. The initiative involved identifying gaps in the quality of services, introducing new tools, improving the work capacity of health care workers through training and mentorship sessions, strengthening laboratory and referral services, and implementing mandatory TB screening of all patients attending health facilities. Supervisors and facility representatives were identified in each intervention facilities. These focal persons were tasked with following the implementation of the toolkit and QI measures with interventions organized across four main strategies.

1. Increasing access to TB services in health facilities: this included raising the index of TB suspicion among health care providers during clinical meetings; continuing medical education, on-the-job mentorship, and supportive supervision; implementing symptomatic screening for TB in all patients presenting to the facility; strengthening referral and linkages within and outside the health facilities; and performing contact tracing to ensure the use of presumptive registers and cascade diagnostic algorithms.
2. Improving the organization and management of TB case-finding activities: this included the sensitization of health facility managers and securing their commitment to improving the quality of TB services, the identification of TB focal persons in each health facility, and holding quarterly meetings among interdisciplinary teams to facilitate the monitoring of TB work plans and strategic problem solving.
3. Improving access to TB diagnosis in health facilities: this included improved sputum sample collection and handling, expanded TB diagnostic services, and strengthened referrals of presumptive and confirmed TB cases within facilities.
4. Strengthening health facilities outreach services to increase access to TB care: health facilities were encouraged to perform community-based activities to improve TB case-finding, including efficient handling of community referrals, follow-up of TB clients who missed appointments, orientations for local leaders and traditional healers, and the sensitization of communities to TB signs and symptoms.

### 2.4. Outcome

The outcome was TB case notifications at 3 months.

### 2.5. Data Management and Analysis

Number of TB case notifications per quarter from all facilities was collected using structured forms and compared with DHIS-ETL. These were entered into Excel for analysis. The data were analysed using Excel and presented as TB case notifications per 100,000 people.

### 2.6. Ethical Considerations

This evaluation was conducted under routine program implementation using programme collected routine data. No individual data or personal identifiers were collected for this evaluation.

## 3. Results

A 52% cumulative increase in the notification of TB cases was recorded at the 10 intervention sites between Q2 of 2016 (pre-QI implementation) and Q2 of 2017, with 8 out of 10 intervention sites reporting increases in their quarterly TB case notifications, as shown in Table 1 below.

A 1% cumulative decrease in the notification of TB cases was recorded at the 10 control sites between Q2 of 2016 (pre-QI implementation) and Q2 of 2017, with 50% of control sites reporting decreases in their quarterly TB case notifications, as shown in Table 2 below.

**Figure 1:**
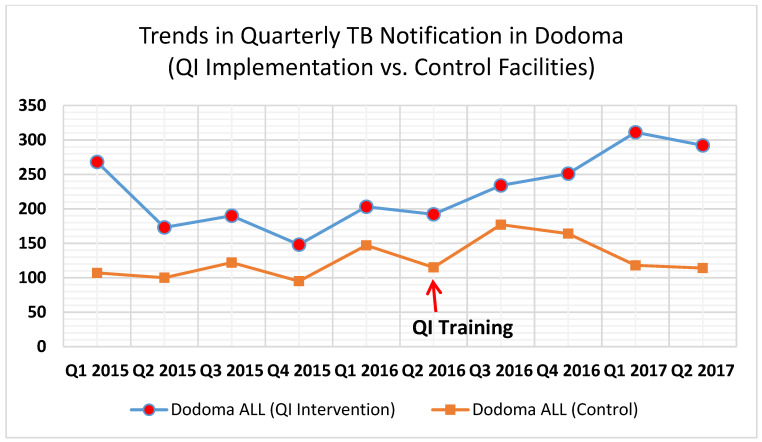
Trends in quarterly tuberculosis case notification in the quality improvement initiative implementation vs. control facilities, Dodoma Region, Tanzania, 2015–2017. QI = quality improvement model.

Among the 10 intervention sites in the Dodoma Region, the pilot registered a 52% increase in TB case notifications in Q1 of 2017 compared with Q1 of 2016 and, similarly, a 52% increase in Q2 of 2017 compared with Q2 of 2016 (Figure 1).

**Figure 2:**
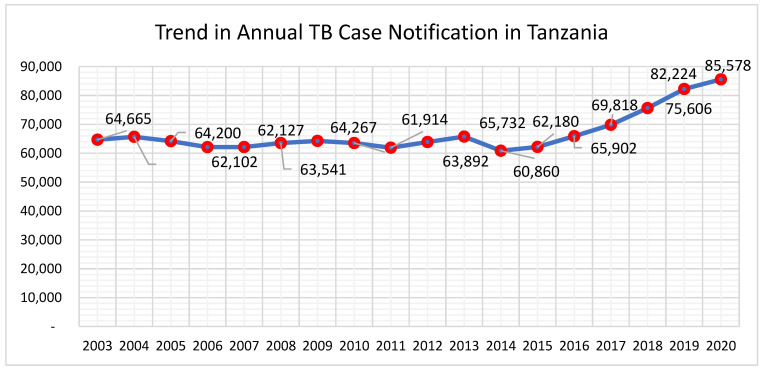
Trend in annual tuberculosis case notification between pre-quality improvement initiative implementation (2014–2015) and the initial pilot and subsequent scale-up of the initiative (July 2016–2017), Tanzania.

After 18 months of implementation, the national TB case notifications increased from 62,180 in 2015 to 85,578 in 2020, as shown in Figure 2, with an average of 4919 additional cases annually, reported between 2017 and 2020.

## 4. Discussion

Overall, the QI initiative contributed to an increase in TB case notifications in the Dodoma region. This is in line with similar initiatives, including the QI-guided active case-finding intervention in northern Uganda, whereby TB case notifications in the intervention districts increased by 30%, i.e., from 171 to 223 per 100,000 people [16,20], and in Kenya, where they employed a behaviour change wheel to aid QI of paediatric TB intervention design and implementation to improve TB case detection in hospitalized children. There were no positive changes in the ‘control facilities’ where routine services were provided. All facilities showed decreases in TB case notifications from baseline.

Following our successful pilot at the subnational level, the QI initiative was rolled out to 1280 additional health care workers in 16 regions, 48 districts, and 530 health facilities between July 2017 and October 2018 [19]. These facilities were purposefully selected to include all regional and district hospitals, as well as health centres and dispensaries that served large catchment areas. Intensive mentoring and supportive supervision were conducted through monthly peer-support and peer mentor groups; technical assistance was provided by TB consultants and the national QI team. Monitoring of QI TB activities was incorporated into all routine supportive supervision and quarterly regional review meetings. Furthermore, policy changes were made to ensure mandatory TB screening in all units of a health facility, prioritizing risk groups; the appointment of a focal person with a clear role of finding missing cases through a decentralized TB services at the health facility; and the integration of a TB focal person and a TB agenda into the health facility quality improvement team (QIT).

However, the challenges encountered during implementation, including inadequate staffing, staff turnover, and reshuffling of health care workers trained in TB case-finding approaches, resulted in the need for repeated skill building and mentorship. Furthermore, there was a slow uptake in QI tools by an already overburdened staff and a lack of fully functional diagnostic tools in some facilities. 

While we acknowledge that the purposeful selection of QI intervention facilities may have created a source of bias, the QI initiative demonstrated how improving the quality of TB interventions could lead to a positive change in TB case notifications. 

## 5. Conclusions

This QI initiative has the potential to support a long-term comprehensive approach to ending TB and to improve the quality of the foundations of the health care system. This initiative sets a reliable pace for health facilities to efficiently respond to and manage TB case-finding interventions put into action. Tanzania’s experience with implementing QI interventions could serve as a model for improving TB case notifications in other settings.

## Figures and Tables

**Table 1:** Quarterly TB case notification in 10 intervention facilities in Dodoma (Q1 of 2016–Q2 of 2017), Tanzania.

Name of Health Facility	Q1 of 2016 n	Q2 of 2016 n	Q3 of 2016 n	Q4 of 2016 n	Q1 of 2017 n	Q2 of 2017 n	Change between Q2 of 2016 to Q2 of 2017 * (%)
Dodoma Regional Hospital	78	80	95	118	138	113	41
Hombolo Health Centre	3	5	3	7	5	15	200
Makole Health Centre	46	35	37	36	68	53	51
Mkonze Health Centre	2	4	4	0	2	7	75
Mvumi Mission Hospital	14	22	50	41	34	53	141
Chamwino Health Centre	30	19	8	12	13	8	−58
Mpwapwa District Hospital	13	17	23	16	31	26	53
Kibakwe Health Centre	8	3	10	5	1	7	133
Chipogoro Health Centre	2	5	3	11	13	9	80
Itiso Dispensary	8	2	1	5	6	1	−50
Dodoma Region (all facilities)	204	192	234	251	311	292	52

* Trend from Q2 of 2016, just prior to the implementation of the QI initiative in July 2016, to Q2 2017, the quarter after the completion of the pilot QI initiative implementation, in all 10 facilities in the Dodoma Region, Tanzania. QI = quality improvement.

**Table 2:** Quarterly TB case notification in 10 control facilities in Dodoma (Q1 of 2016–Q2 of 2017), Tanzania.

Facility	Q1 of 2016	Q2 of 2016	Q3 of 2016	Q4 of 2016	Q1 of 2017	Q2 of 2017	Change between Q2 of 2016 to Q2 of 2017 * (%)
Bahi Health Centre	8	16	11	9	21	17	6
Kigwe Health Centre	4	2	8	4	5	4	100
Mundemu Health Centre	5	8	5	5	2	3	−63
Kondoa District Hospital	48	32	48	64	35	29	−9
Busi Health Centre	4	4	9	3	4	5	25
Mnenia Health Centre	3	6	5	4	3	1	−83
Kongwa District Hospital	22	16	36	31	15	20	25
Kibaigwa Health Centre	21	6	16	18	13	19	217
Mlali Health Centre	12	17	28	13	15	11	−35
Hamai Health Centre	20	8	11	13	5	5	−38
Dodoma ALL (Control)	147	115	177	164	118	114	−1

* Trend from Q2 of 2016, just prior to the implementation of the QI initiative in July 2016, to Q2 2017, the quarter after the completion of the pilot QI initiative implementation, in all 10 facilities in the Dodoma Region, Tanzania. QI = quality improvement.

## Data Availability

The data used are collected routinely by the NTLP with a case-based database in DHIS2-ETL and are analysed annually for decision making and planning. The programme annual reports with analysed data are also available for use.
